# The Potential Equivalents of *TET2* Mutations

**DOI:** 10.3390/cancers13071499

**Published:** 2021-03-24

**Authors:** Sergiu Pasca, Ancuta Jurj, Mihnea Zdrenghea, Ciprian Tomuleasa

**Affiliations:** 1 Department of Hematology, Iuliu Hatieganu University of Medicine and Pharmacy, 400012 Cluj Napoca, Romania; pasca.sergiu123@gmail.com (S.P.); mzdrenghea@umfcluj.ro (M.Z.); ciprian.tomuleasa@umfcluj.ro (C.T.); 2 Medfuture Research Center for Advanced Medicine, Iuliu Hatieganu University of Medicine and Pharmacy, 400349 Cluj Napoca, Romania; 3 Department of Hematology, Ion Chiricuta Clinical Cancer Center, 400124 Cluj Napoca, Romania; 4 Research Center for Functional Genomics, Biomedicine and Translational Medicine, Iuliu Hatieganu University of Medicine and Pharmacy, 400337 Cluj-Napoca, Romania

**Keywords:** TET2, mutations, equivalent, expression

## Abstract

**Simple Summary:**

In acute myeloid leukemia (AML) *TET2* mutations have been observed to be mutually exclusive with *IDH1*, *IDH2*, and *WT1* mutations, all of them showing a similar impact on the transcription profile. Because of this, it is possible that *TET2/IDH1/2/WT1* mutated AML could be considered as having similar characteristics between each other. Nonetheless, other genes also interact with TET2 and influence its activity. Because of this, it is possible that other signatures exist that would mimic the effect of *TET2* mutations. Thus, in this review, we searched the literature for the genes that were observed to interact with TET2 and classified them in the following manner: transcription alteration, miRs, direct interaction, posttranslational changes, and substrate reduction.

**Abstract:**

TET2 is a dioxygenase dependent on Fe^2+^ and α-ketoglutarate which oxidizes 5-methylcytosine (5meC) to 5-hydroxymethylcytosine (5hmeC). TET proteins successively oxidize 5mC to yield 5-hydroxymethylcytosine (5hmC), 5-formylcytosine (5fC), and 5-carboxylcytosine (5caC). Among these oxidized methylcytosines, 5fC and 5caC are directly excised by thymine DNA glycosylase (TDG) and ultimately replaced with unmethylated cytosine. Mutations in *TET2* have been shown to lead to a hypermethylated state of the genome and to be responsible for the initiation of the oncogenetic process, especially in myeloid and lymphoid malignancies. Nonetheless, this was also shown to be the case in other cancers. In AML, *TET2* mutations have been observed to be mutually exclusive with *IDH1*, *IDH2*, and *WT1* mutations, all of them showing a similar impact on the transcription profile of the affected cell. Because of this, it is possible that *TET2/IDH1/2/WT1* mutated AML could be considered as having similar characteristics between each other. Nonetheless, other genes also interact with TET2 and influence its effect, thus making it possible that other signatures exist that would mimic the effect of *TET2* mutations. Thus, in this review, we searched the literature for the genes that were observed to interact with TET2 and classified them in the following manner: transcription alteration, miRs, direct interaction, posttranslational changes, and substrate reduction. What we propose in the present review is the potential extension of the *TET2/IDH1/2/WT1* entity with the addition of certain expression signatures that would be able to induce a similar phenotype with that induced by TET2 mutations. Nonetheless, we recommend that this approach be taken on a disease by disease basis.

## 1. Introduction

TET2 is a dioxygenase dependent on Fe^2+^ and α-ketoglutarate which oxidizes 5-methylcytosine (5meC) to 5-hydroxymethylcytosine (5hmeC). TET proteins successively oxidize 5mC to yield 5-hydroxymethylcytosine (5hmC), 5-formylcytosine (5fC), and 5-carboxylcytosine (5caC). Among these oxidized methylcytosines, 5fC and 5caC are directly excised by thymine DNA glycosylase (TDG) and ultimately replaced with unmethylated cytosine. This mechanism not only provides a pathway for removing the methyl mark from cytosines but also provides a mechanism for generating 5hmeC marks. The 5hmeC marks also play an important role in being recognized by certain epigenetic readers leading to further alterations to the expression profile [1]. This mark (5hmeC) has been observed to be altered in aging, cancer, cell differentiation, and other diseases and biological processes [2]. Added to this, TET2 is also involved in the oxidation of 5meC to 5hmeC in RNA species [3].

The function of TET2 was revealed to be of high importance especially regarding myeloid and lymphoid cells. For example, TET2 is essential for the differentiation of monocytes [4]. Added to this, *TET2* deletion increases the number of immature myeloid cells (assessed through the c-Kit positive and Lin negative markers) in the bone marrow of murine models. This was one of the arguments that showed the importance of TET2 in myeloid maturation [5]. Moreover, inhibition of the conversion of 5meC to 5hmeC induced experimentally through TET2 alteration leads to altered granulopoiesis and erythropoiesis [6]. The effect that TET2 has on erythropoiesis was also observed in a zebrafish model, having a similar role as in mammals [7].

A more direct link between the alteration of this enzyme and the development of certain diseases has been based on the fact that mice lacking *TET2* frequently develop myeloid malignancies [8]. More than this, tumor development, in general, is associated with a reduction in 5hmC marks. This is correlated with reduced TET2 activity in lung, prostate, pancreatic, liver, and breast cancers when compared to the surrounding tissue [9]. Added to this, TET2 is also important in the development of angioimmunoblastic T-cell lymphoma (AITL), being one of the first mutations that appear in this disease [10,11]. Interestingly, *TET2* alterations also induce instability of FOXP3, leading to an important tendency for autoimmunity in murine models [12,13]. What has to be mentioned is the fact that AITL is generally associated with autoimmune events, especially autoimmune hemolytic anemia. The association of AITL with autoimmune events is also in accordance with the fact that the cell from which AITL develops is represented by the T-follicular helper cell, which is implicated in stimulating various processes of the immune systems, including antibody production [14]. Even though hematologic malignancies present the most interest regarding *TET2* mutations, the literature also contains data regarding the presence of *TET2* mutations in other malignancies. Some examples are the field of sarcomas, specifically of chondrosarcoma [15], and glioma where it has been observed to be a driver in the absence of *IDH1/2* mutations [16].

Because TET2 is at the center of these mechanisms for epigenetic modifications, the scientific community presented a high interest regarding this enzyme. Nonetheless, the molecules that interact with TET2 are still presenting a high interest in the scientific community as alterations in some of these can mimic the effect of *TET2* mutations. Thus, in the present review, we will discuss the genes that influence TET2 activity potentially mimicking the effects of *TET2* mutations. This is important as it would represent a step to considering some signatures as having the same effect in a certain malignancy and, thus, considering them as having similar effects to that of *TET2* mutations.

## 2. Classic Gene Mutations Mimicking *TET2* Loss

Some of the best-studied mutations that influence TET2 activity are represented by mutations in ***IDH1*** and ***IDH2***. In this case, mutations in *TET2*, *IDH1*, and IDH2 are mutually exclusive in AML. Normally, both IDH1 (cytoplasmic isoenzyme) and *IDH2* (mitochondrial isoenzyme) catalyze the conversion of isocitrate to α-ketoglutarate. Mutations in these enzymes generally occur in three hotspots (R132 for IDH1 and R140 and R172 for IDH2) and are all gain-of-function mutations. More specifically, these mutations switch IDH1/2 from converting isocitrate to α-ketoglutarate to, instead, to converting α-ketoglutarate to 2-hydroxyglutarate. The latter compound (2-hydroxyglutarate) is sterically similar to α-ketoglutarate and acts as a competitive inhibitor to α-ketoglutarate-dependent enzymes [17]. Of those, an important α-ketoglutarate dependent enzyme is TET2, with *IDH1/2* mutations being able to mimic the effect of *TET2* mutations leading to a similar transcriptomic profile as we have previously shown [18].

These mutations are implicated in oncogenesis by impairing cell differentiation and, thus, increasing the number of cancer stem cells in a variety of malignant diseases including AML [19], glioblastoma [20], sarcoma [21], cholangiocarcinoma [22], and others [23]. Interestingly, these mutations appear early in the process of oncogenesis [23]. Because of the role that *IDH1/2* mutations have in this process, inhibitors have been developed to target these mutations with the best-known ones being represented by ivosidenib and enasidenib. These inhibitors have been used in the treatment of *IDH1* and *IDH2* mutated AML and have been approved by the Food and Drug Administration [24,25]. Nonetheless, it was proposed that these mutations can also be targeted in other cancers [26]. Added to this, because of the frequency of *TET2* and *IDH1/2* mutations in lymphomas developed from a T-follicular helper cell (e.g., AITL, peripheral T-cell lymphoma not otherwise specified) drugs targeting epigenetic alterations were also used in clinical trials. In this case, clinical trials have shown the efficacy of azacitidine combined with romidepsin in inducing a response in these diseases. This adds arguments to the fact that these lymphomas are heavily dependent on mutations in epigenetic modifiers [27,28].

The gain-of-function *IDH1/2* mutations that convert α-ketoglutarate to 2-hydroxyglutarate, a competitive inhibitor for the TET2 enzyme as well as mutual exclusivity of *IDH1* and *TET2* mutations in certain cancer types, particularly in AML, lead to the widely accepted concept that mutant *IDH1* modulates tumorigenesis through the effect on TET2 activity to alter the tumor epigenetic landscape. However, the lack of complete phenotypic overlap between IDH1 mutant and TET2 mutant cancers suggests that IDH1 can modulate leukemogenesis independently of TET2. For instance, unlike *TET2* knock-out mice, mice harboring *IDH1* mutations did not show any significant alterations in the proportion of hematopoietic progenitors in the bone marrow, and their bone marrow cells did not show an advantage over wild-type cells in competitive repopulation assays. More importantly, progression to acute leukemia was not reported in *IDH1/2* mutant mice [29,30,31].

Furthermore, while TET2 mutations are more frequent in chronic myelomonocytic leukemia (CMML) than in AML, IDH1/2 mutations are more common in AML than in CMML [32,33]. Unlike TET2 mutations, IDH1/2 mutations are quite rare in clonal hematopoiesis [34,35]. As mentioned before, *IDH1/2* and *TET2* mutations are not mutually exclusive but rather coexist in a large fraction of patients in some cancers such as AITL. Mutant *IDH1* can broadly inhibit the activities of a number of enzymes including approximately 60 α-ketoglutarate-dependent enzymes and is also shown to downregulate ATM and thus alter DNA repair and sensitivity to DNA damage independent of TET2 [36,37]. Thus, it is also likely that *TET2* and *IDH1/2* mutations can have at least some differential tumorigenic effects, even if they partly share a common oncogenic mechanism.

**Figure 1:**
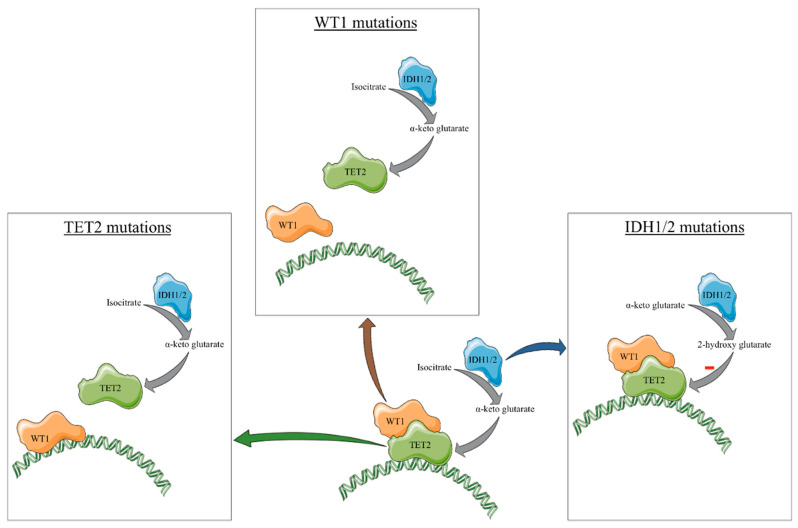
Overview of the mechanisms through which *IDH1*, *IDH2*, and *WT1* mutations induce, in some cases, transcriptional profiles similar to those induced by *TET2* mutations.

Other gene mutations that can be observed to present mutual exclusivity with *TET2/IDH1/2* in AML are represented by mutations in ***WT1***. WT1 physically interacts with TET2 and is involved in the action that TET2 has at the DNA level. Mutations at the level of this gene lead to the formation of truncated or nonfunctional forms of WT1 which are either degraded earlier at mRNA level [38] or lack the ability to aid the interaction between TET2 and DNA. Because of this interaction, mutations in *WT1* induce a similar effect to that of *TET2* mutation [39]. More than this, we have shown that mutations in *WT1* are associated with similar methylation and transcriptomic profiles to that of *TET2/IDH1/2* mutations in the case of AML [18]. In the same article, we proposed that *TET2/IDH1/2/WT1* mutated AML to be considered as being similar to each other. Nonetheless, further data is needed to support this evidence. Conversely, compared to *TET2/IDH1/2* mutations, which are generally present as initiating mutations in AML and have a relatively stable presence, *WT1* mutations are considered to be secondary events in AML and are much less stable [40]. However, whether *WT1* and *TET2* mutations share similar transcriptional targets or whether they cause similar hematopoietic abnormalities including aberrant hematopoiesis or leukemogenesis has not been directly assessed, particularly in in vivo settings. An overview of the effects of *TET2/IDH1/2/WT1* mutations on the function of TET2 is represented in Figure 1.

## 3. Interactions within the *TET2/IDH1/2/WT1* Mutated Cells

Another important step to be taken is to determine how *TET2/IDH1/2/WT1* mutations interact with other mutations or gene expressions to alter the global transcriptomic profile.

In this direction, we studied how *TET2/IDH1/2/WT1* mutations affect the mRNA associations of the *RUNX1* mRNA. In our study, we observed that *TET2/IDH1/2/WT1* mutated AML cases are associated with an increase in negative correlations between the *RUNX1* mRNA and other mRNAs. More than this, when assessing the processes in which the negatively correlated genes are involved in we observed the presence of several genes involved in myeloid lineage activity. Because of this, this may be one of the changes that occur in *TET2/IDH1/2/WT1* mutated AML that leads to the generation of an immature phenotype of the AML clone [41]. Nonetheless, more studies are needed to better assess the interaction between RUNX1 and TET2.

Another example in this direction is represented by the co-occurrence between *DNMT3A* and *TET2* mutations, which, in theory, should have opposing effects. Interestingly the double knockout of *TET2* and *DNMT3A* showed that the DNA methylation profile has regions that are cooperatively, competitively, and independently influenced by these alterations with KLF1 being an important transcription factor that is upregulated when both *TET2* and *DNMT3A* are deleted [42]. Thus, *TET2/IDH1/2/WT1* mutations might induce different methylation and transcriptomic profiles when associated with *DNMT3A* alterations.

## 4. Other Equivalents of *TET2* Mutations

**Figure 2:**
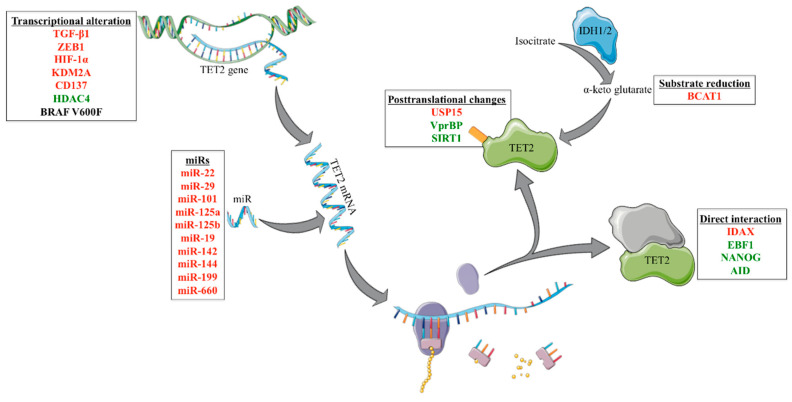
Overview of the genes implicated in the regulation of TET2 activity except *for IDH1*, *IDH2*, and *WT1* mutations. In the red font, we depicted genes that, when upregulated, would reduce TET2 activity, while, with green, we depicted genes that, when downregulated, would reduce TET2 activity. An exception to this rule is *BRAF*, in which case we discussed the V600F mutation and not an expression alteration. Additionally, in the case of direct interaction, we also depicted synergy as green.

*IDH1*, *IDH2*, and *WT1* mutations have been thoroughly studied and important evidence has been built to argue for their influence on TET2. Nonetheless, there are still other genes that interact with TET2 and have the potential to alter its activity. Not necessarily gene mutations, but alterations in expression or posttranslational modifications in some proteins might heavily influence TET2. Because of this, we consider that it would be possible for additional signatures to exist that offer a phenotype similar to that of *TET2* mutations. Further, we will discuss the molecules that interact with TET2. We will refer to these interactions as transcription alteration, miRs, direct interactions, posttranslational changes, and substrate reduction. An overview of the different molecules observed in the literature that we will be further discussing was represented in Figure 2.

### 4.1. Transcription Alteration

In melanoma, **TGF-β1** determines the recruitment of DNMT3A to the *TET2* gene locus, methylation of this gene, and subsequent silencing of *TET2* expression, which induces an increase in the process of epithelial-mesenchymal transition [43]. Because this process is dependent on DNMT3A, an interesting research direction would be to delete *DNMT3A* and determine if this phenomenon is still present. Conversely, in diabetic nephropathy, TET2 acts as a regulator of TGF-β1 by initiating the process of demethylation at the *TGF-β1* regulatory regions and increasing its expression [44]. Because of this, it is possible that TGF-β1 acts as an indirect negative regulator for its own expression. Nonetheless, this hypothesis has to be validated in the same disease (e.g., AML) for it to reveal a reliable signaling pathway.

In glioma, **ZEB1** binds to the *TET2* promoter repressing its expression. The reduction in *TET2* expression is associated with an increase in glioma invasion and growth [45].

In melanoma cells, **HIF-1α** downregulation increases TET2 expression both considering the mRNA and protein levels [46].

In breast cancer **KDM2A** interacts with RelA and acts on the promoter of *TET2*, inhibiting *TET2* expression. This is in accordance with the fact that *KDM2A* knock-down is associated with *TET2* overexpression, while *KDM2A* ectopic expression suppresses *TET2* transcription. More than this, *KDM2A* knock-down is associated with an increase in genomic 5hmeC marks [47].

**CD137** activation decreases the expression of *TET2* in endothelial cells. More than this, CD137 activation also inhibits the transfer of exosomes loaded with TET2 from endothelial cells to vascular smooth muscle cells. Because of this, CD137 decreases the anti-inflammatory protection offered by TET2 and increases the process of neointimal formation. It has to be mentioned that this study did not assess promoter binding of any molecules activated by CD137 but only the expression patterns [48].

In myelodysplastic syndrome (MDS) and AML cells, **HDAC4** inhibition leads to the downregulation of TET2 and the subsequent decrease in the abundance of 5hmeC marks [49].

In colon cancer, ***BRAF*** V600F was associated with the downregulation of TET2 and subsequent DNA hypermethylation [50].

### 4.2. miRs

miRs have also been observed to target TET2 mRNA, thus influencing the abundance of TET2 that can perform its function. Using the miRTargetLink Human software [51], we observed that there were 8 miRs presenting strong evidence, 9 presenting weak evidence, and 89 predicted of interacting with *TET2*. The miRs presenting strong evidence were represented by: miR-22-3p, miR-101-3p, miR-29a-3p, miR-29b-3p, miR-29c-3p, miR-26a-5p, miR-7-5p and miR-125b-5p. The miRs presenting weak evidence were represented by: miR-335-5p, miR-484, miR-92a-3p, miR-5011-5p, miR-1277-5p, miR-3924, miR-190-3p, miR-4775 and miR-590-3p. We further chose to assess the miRs presenting strong evidence by introducing them in miRNET [52] using bone marrow as the selected tissue and miRTarBase v8.0 [53] as the database for miR targets. Using this algorithm, the network generated contained 2117 genes and 2804 edges. Aside from *TET2*, which was targeted by all of the assessed miRs, we also find it important to mention that *DNMT3A* was targeted by 4 of these miRs, showing that TET2 and DNMT3A do not necessarily have to act in antagonism. Interestingly, we also found SIRT1 to be targeted by 2 of these miRs. This is important as SIRT1 deacetylates the c-terminus portion of TET2 and increases its activity [54]. We also assessed the KEGG pathways that are enriched considering these genes and we would like to mention the following KEGG [55] terms preceded by the number of genes associated with that term: 24 genes for AML, 35 for glioma, 27 genes for apoptosis, 28 genes for the p53 signaling, 21 genes for the mTOR signaling pathway, 44 genes for the WNT signaling pathway and 29 genes for the TGF-β signaling pathway.

**MiR-22** was shown to be upregulated in MDS and to be associated with a reduction in the global abundance of the 5hmeC mark. This was caused by the fact that miR-22 targets *TET2* and reduces its abundance. Transgenic mice overexpressing miR-22 in the hematopoietic compartment present hematopoietic stem cells that have an increased ability to undergo self-renewal, but a reduced ability to differentiate, thus setting the basis for leukemic transformation [56]. Added to this, miR-22-3p is also implicated in the process of vascular smooth muscle cell differentiation by impairing *TET2* translation. More than this, the circular RNA circMAP3K5 can sponge miR-22-3p, increasing the effects of TET2, which mediates vascular smooth muscle cell differentiation [57].

**MiR-29** targets *TET2* and increases the WNT/β-catenin leading to reduced efficiency in the reprogramming of fibroblasts to stem cells. An additional argument of this effect is that, naturally, in the process of reprogramming, miR-29 is downregulated [58]. In the case of porcine early embryo development, miR-29b was observed to influence DNA methylation by targeting *TET1/2/3* and *DNMT3A/B*, thus potentially having an effect both on demethylation as well as on de novo methylation [59].

In human umbilical vein endothelial cells, the upregulation of **miR-101-3p** is associated with increased production of reactive oxygen species and the upregulation of NFκβ. These effects are mediated through the repression of *TET2* translation by miR-101-3p, which thus reduces TET2 anti-inflammatory activity [60].

In an MLL-AF9 AML murine model, **miR-125b** targets *TET2* leading to the upregulation of VEGF-A and subsequent increase in AML cell proliferation and apoptosis reduction [61]. In giant cell bone tumors, miR-125a upregulation is associated with tumor growth and recurrence. This effect is caused by the inhibition of TET2, which increases the IL-17A expression [62]. MiR-125a-5p is also implicated in the pyroptosis of endothelial cells when stimulated by oxidized low-density lipoproteins. This process was determined to be caused by the miR-125a-5p mediated inhibition of *TET2* that determined alterations at several levels including DNA methylation, mitochondrial processes, and the production of reactive oxygen species. These effects were further associated with overexpression of *IL-1β* and *IL-18*, cytokines implicated in the inflammatory process [63].

We have to mention that we found miRs in the literature that did not appear in the searched database which we will be further discussing.

**MiR-19a-5p** targets *TET2* in glioblastoma inducing proliferation and metastasis. Interestingly, miR-19a-5p was shown to be sponged by the lncRNA AC016405.3 which repressed the glioblastoma cells by reestablishing the expression of *TET2* [64].

In Treg cells from models of type 1 diabetes, **miR-142-3p** targets *TET2*, thus affecting the activity of FOXP3 and leading to the reduction of the activity of Treg cells [12].

In bone marrow stem cells from patients with aplastic anemia, **miR-144-3p** inhibits osteogenic differentiation by targeting *TET2* [65].

In osteoblasts from ovariectomized mice, **miR-199a-5p** was decreased compared to the controls. This observation was associated with *TET2* upregulation and a decrease in osteoblast differentiation. Conversely, miR-199a-5p upregulation and subsequent *TET2* downregulation were associated with better bone development and upregulation of genes involved in the osteogenesis process [66].

In breast cancer cells, **miR-660-5p** targets *TET2* and is associated with a higher stage and an increased vascular invasion in breast cancer patients. In in vitro assays, these results were confirmed by showing that miR-660-5p downregulation was associated with a reduced invasion and an increased apoptosis rate. More than this, aside from the downregulation of *TET2*, these effects are also mediated through the increased activity of the PI3K/AKT/mTOR signaling pathway [67].

### 4.3. Direct Interaction

***IDAX*** was originally encoded within an ancestral *TET2* gene that underwent a chromosomal gene inversion during evolution, thus separating the TET2 CXXC domain from the catalytic domain. Moreover, it has been shown that IDAX interacts with the catalytic domain of TET2. It also has to be mentioned that IDAX upregulation is associated with TET2 downregulation and caspase activation. Because of this, it is thought that IDAX can interact with DNA localized TET2 and induce the degradation of TET2 [68]. Because of this, the *IDAX* expression level after a certain cutoff might be equivalent to *TET2* mutations.

**EBF1** interacts with TET2 in multiple cancers like glioma, AML, and chondrosarcoma. Added to this, EBF1 co-localizes with TET2 in hypermethylated loci of the DNA [69]. EBF1 also induces specific chromatin modifications in B-cell development. These changes occur in processes like VDJ rearrangement and antigen stimulation [70]. Conversely, EBF1 is localized in hypomethylated regions in the case of breast cancer [71]. Because of this, EBF1 might influence the effect that TET2 has on the DNA, with alterations in EBF1 also having a potential impact on the activity of TET2.

**NANOG** can physically interact with TET2 with an important role in the mechanisms of pluripotency of stem cells. It has been observed that the knockout of TET2 diminishes the effect of NANOG because of the direct interaction it has with TET2 in normal circumstances. More than this, co-expression of NANOG and TET2 increases stem cell pluripotency. A further argument to the interaction between TET2 and NANOG is represented by the fact that they co-localize at various genomic loci [72].

In diffuse large B cell lymphoma, **activation-induced cytidine deaminase (AID)** cooperates with TET2 binding to the *FANCA* promoter and thus increasing the expression of *FANCA*. Because of these results, the authors of the study proposed the combined use of AID and TET2 depletion in addition to bortezomib to better manage diffuse large B cell lymphoma [73].

### 4.4. Posttranslational Changes

An important part of protein regulation is represented by ubiquitylation which classically has been described as a way for protein degradation. Nonetheless, it has been shown that ubiquitylation can regulate different pathways, being involved as a posttranslational modification that influences signal transmission [74]. In the case of TET2, two proteins have been studied in conjunction with TET2, namely **VprBP** and **USP15**. VprBP is implicated in the last step of ubiquitylation leading to the transfer of ubiquitin from E2 to the substrate. This is important in our case because it has been shown that this enzyme monoubiquitylates TET2 at the position K1299 promoting its binding to chromatin. More than this, it has been observed that the lack of this signal leads to a reduction in the activity of TET2 with a subsequent reduction of 5hmC marks in the genome. VprBP has been shown to bind to the cysteine-rich domain of TET2, even if TET2 is present in a truncated form, lacking its active site. This shows that VprBP binding is independent to TET2 and the catalytic activity of TET2 [75]. Conversely, USP15 deubiquitylates TET2 K1299, negatively regulating TET2 activity. The deletion of USP15 in melanoma cells increases TET2 activity. This activates the IFN/JAK/STAT signaling pathway in the T-cells from the tumor microenvironment through the increase in the IFNγ secretion [76].

**SIRT1** was observed to inhibit MDS maintenance in a TET2 dependent manner by deacetylating the c-terminus end of TET2. This change determines the normal maintenance of hematopoietic stem cells in stress conditions. Conversely, SIRT1 downregulation was observed in the case of MDS. The authors of the study also proposed the use of SIRT1 activation as a therapeutic approach in MDS as it would enhance the ability of stem cells to resist in the context of a pro-inflammatory microenvironment [54].

### 4.5. Substrate Reduction

One manner through which other molecular traits could lead to a similar phenotype to that of *TET2/IDH1/2/WT1* mutant cells is through the reduction of the substrates of TET2. In this direction, **BCAT1** upregulation in AML cells determines a reduction in α-ketoglutarate, which mimics the effect of *IDH1/2* mutations [77].

## 5. Concluding Remarks and Future Perspectives

Thus, the knowledge about the molecular species that influence TET2 activity has greatly increased from that of *IDH1*, *IDH2*, and *WT1* mutations to several factors influencing TET2 expression, translation, posttranslational marks, substrate, and the direct effect that TET2 has. Nonetheless, it has to be mentioned that *IDH1*, *IDH2*, and *WT1* were assessed as gene mutations whereas most of the other molecular species mentioned here take into consideration their expression level. This is important as gene mutations are more reliably detected compared to expression levels which could be influenced by study errors. More than this, in this review we presented the interactions that TET2 has across different malignancies, cardiovascular diseases, and other conditions. Considering that there might be a difference between the effect that *TET2* and *IDH1/2* mutations have between AML and AITL, the present review has to be taken with a certain caution and be used to orient at some genes and then validate them on the disease of interest as having a similar effect to that of *TET2* mutations.

From a clinical standpoint, mutations are easier to be assessed in the clinical scenario, several hematology clinics having mutational panels available that are tailored to each disease subset, whereas expression of different genes is rarely if ever, assessed in the current clinical practice, implementing the proposed equivalents difficult at this point in time. Nonetheless, with the advancement of clinical assessments, there might be a point at which routine gene expression could be done in the clinical scenario.

What we propose in the present review is the potential extension of the *TET2/IDH1/2/WT1* entity with additional expression signatures that would be able to induce a similar phenotype to the cell of interest. Nonetheless, we recommend that this approach be taken on a disease by disease basis.
